# Investigation of MYST4 histone acetyltransferase and its involvement in mammalian gametogenesis

**DOI:** 10.1186/1471-213X-7-123

**Published:** 2007-11-02

**Authors:** Serge McGraw, Guillaume Morin, Christian Vigneault, Pierre Leclerc, Marc-André Sirard

**Affiliations:** 1 Département des Sciences Animales, Centre de Recherche en Biologie de la Reproduction, Université Laval, Québec, Canada; 2 Département d'Obstétrique/Gynécologie, Centre de Recherche en Biologie de la Reproduction, Université Laval, Québec, Canada

## Abstract

**Background:**

Various histone acetylases (HATs) play a critical role in the regulation of gene expression, but the precise functions of many of those HATs are still unknown. Here we provide evidence that MYST4, a known HAT, may be involved in early mammalian gametogenesis.

**Results:**

Although *MYST4 *mRNA transcripts are ubiquitous, protein expression was restricted to select extracts (including ovary and testis). Immunohistochemistry experiments performed on ovary sections revealed that the MYST4 protein is confined to oocytes, granulosa and theca cells, as well as to cells composing the blood vessels. The transcripts for *MYST4 *and all-*MYST4*-isoforms were present in oocytes and in *in vitro *produced embryos. In oocytes and embryos the MYST4 protein was localized in both the cytoplasm and nucleus. Within testis sections, the MYST4 protein was specific to only one cell type, the elongating spermatids, where it was exclusively nuclear.

**Conclusion:**

We established that MYST4 is localized into specialized cells of the ovary and testis. Because the majority of these cells are involved in male and female gametogenesis, MYST4 may contribute to important and specific acetylation events occurring during gametes and embryo development.

## Background

In eukaryotic cells, the tightly packed chromatin contained in the nucleus directs fundamental cellular processes. The regulation of chromatin conformation by specific structural proteins and their post-translational modifications have a major influence on transcription, repair, replication and recombination [1-5]. Histones are important for chromatin organization and their residues are constantly targeted by modification enzymes. One of the modifications implicates acetylation of specific lysine residues in core histones (H2A, H2B, H3 and H4). By its ability to remodel chromatin, histone acetylation influences the transcriptional state of chromosomal regions by controlling the accessibility of underlying genes, directly linking this regulatory mechanism with gene activation [6]. Acetylation of histones is also involved in the deposition of free histones onto newly synthesized DNA [reviewed in [7]] and in the replacement of histones by protamines [8]. Because histone acetylases (HATs) and deacetylases (HDACs) are associated with crucial regulatory roles, their dysregulation is often involved in diseases such as cancer [9].

HATs are divided into 3 families: Gcn5/PCAF (general control of amino-acid synthesis 5/p300-CBP-associated factor), p300/CBP (Adenoviral E1A-associated protein/CREB-binding protein) and MYST. Among these families, MYST is more divergent and not as well characterized. This protein family is also different with regard to domain organization, multiprotein complex formation and biological function [10,11]. MYST is an acronym of its four founding members: human MOZ (monocytic leukemia zinc finger protein), yeast Ybf2 (renamed Sas3, for something about silencing 3), yeast Sas2, and mammalian TIP60 (HIV Tat-interacting 60 kDa protein) [11]. MYST4, also called MOZ2 or MORF (monocytic leukemia zinc finger protein-related factor), is a member of the MYST family [12]. *In vitro *studies demonstrated that it preferentially acetylates free histones H2A, H3 and H4, as well as nucleosomal H3 and H4. Alternative splicing variants are found (MORF, MORFα and MORFβ), but their expression, impact and function remain uncharacterized. The name MYST4 is now attributed to the longest of the splicing variant, MORFβ. The sequences composing some of MYST4's domains are similar only to one other MYST family member, MOZ. Both MOZ and MYST4 are involved in leukemogenesis [13-15]. Chromosomal abnormalities found in leukemia patients reveal that *MYST4 *is rearranged and fused with the *CBP *gene [13-15], a translocation also associated with *MOZ *[16]. Additionally MYST4 can interact with RUNX1 (Runt-related transcription factor 1), a recurrent leukemia associated target [17]. In the mouse, its homologue Querkopf is thought to be implicated in cell differentiation in the cerebral cortex by regulating chromatin organization at some point during transcription. The malformations found in the cerebral cortex of mutant *querkopf *mice reveal that the gene is essential for normal embryonic neurogenesis [18]. Its involvement in gametogenesis and early embryogenesis is unknown, however preliminary mRNA studies revealed that *MYST4 *transcripts are present in high amounts in bovine oocytes (S. McGraw, unpublished results) compared to other HATs [19].

Many members of the MYST family have distinct domains and diverse functions, including roles in epigenetic control, transcriptional regulation, DNA replication, DNA repair, chromatin assembly, cell cycle progression and cellular signalling (reviewed in [20]). It has been suggested that MYST4 may also perform some of those functions, although since it has unique domains it may act differently from other MYST members [17]. Structural features found in MYST4 suggest that it could be a HAT with novel properties. However, most functions and characteristics attributed to MYST4 remain hypothetical and are only based on sequence analysis. The lack of proteomic and more thorough *in vivo *data are due to the absence of a functional MYST4 antibody.

In the study presented here, our aim was to characterize bovine *MYST4 *and the combination of all *MYST4 *splicing variants simultaneously (*MORF*, *MORFα*, *MORFβ*) in various tissues, but more specifically in reproductive tissues and gametes. The transcriptional study revealed that *MYST4 *was ubiquitously expressed in bovine somatic tissues. Unlike its mRNA, the MYST4 protein was present in some, but not all, of the eleven somatic tissues tested. In addition, MYST4 could be linked with important events that take place during folliculogenesis, embryo development, and spermatogenesis. *MYST4 *mRNA levels were assessed in oocytes and embryos, as were protein expression and localization. Valuable information was additionally obtained with the localization and expression of MYST4 protein in the ovary and testis.

## Results

### MYST4 Sequence

**Figure 1:**
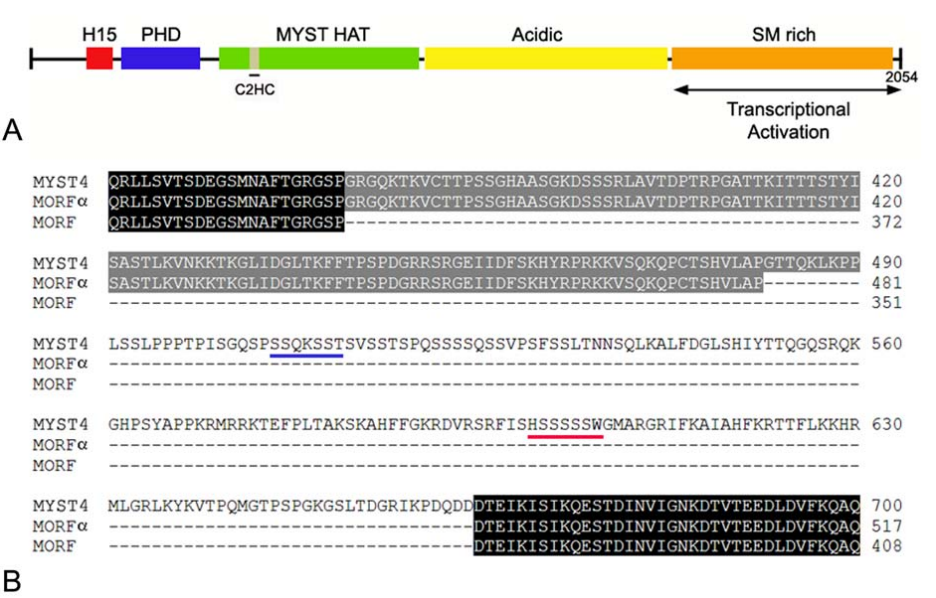
**Representation of Bovine MYST4**. A) Domain organization of MYST4. H15, linker histones H1- and H5-like domain; PHD, plant homeodomain zinc finger; C2HC, zinc finger; MYST HAT, conserved HAT domain characteristic of MYST family members; Acidic, glutamate/aspartate-rich region; SM-rich, serine/methionine-rich domain. MYST4 is composed of 2054 residues in which the N-terminal region and the SM-rich domain encode transcriptional repression and activation domains respectively. B) Schematic illustration of MYST4 showing the alternative MYST4 splicing variants MORF and MORFα. Conserved regions between either 2 or 3 sequences are highlighted in gray and black respectively. Regions used for *MYST4 *primer designs are underlined in blue (forward) and in red (reverse). The number of residues in each sequence is indicated on the right. (Accession numbers: MYST4; [GenBank; XM_867653], MORF; [GenBank; XM_615480], MORFα; [GenBank; XM_880818])

Using polymerase chain reaction on oocyte cDNA, we obtained an amplified fragment of approximately 6600 bp (not shown). Sequencing revealed that it corresponded to the full-length bovine *MYST4 *gene. This splicing variant is the longest of the three MYST4 isoforms and encoded 2054 amino acid residues (Fig. 1A). The three splicing variants contained exactly the same sequences, except that MORF and MORFα are 292 aa and 109 aa shorter than MYST4 (MORFβ) (Fig. 1B). Alignment analysis also revealed that conservation between human and bovine MYST4 was nearly 95%. The human MYST4 sequence has 19 more amino acids, which can be found almost entirely in two glutamic acid-rich areas located in its acidic region. Bovine MYST4 contains various structural domains throughout its coding sequence. The most homologous domains were similar to those found in other proteins such as linker histones H1/H5, PHD zinc fingers and MYST histone acetylases (Fig. 1A). Additionally acidic and Ser/Met-rich regions were found. A transcriptional activation domain was also present in the C-terminal sequence of MYST4.

### MYST4 mRNA in Somatic Tissues

**Figure 2:**
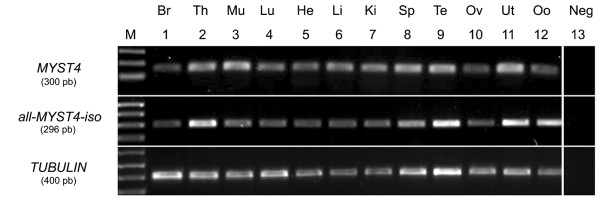
**RT-PCR analysis of *MYST4 *mRNA**. Bovine *MYST4 *and all-*MYST4*-iso mRNA expression was investigated in different tissues. From left to right, Br, brain; Th, thymus; Mu, muscle; Lu, Lung; He, heart; Li, liver; Ki, kidney; Sp, spleen; Te, testicle; Ov, ovary; Ut, uterus; Oo, germinal vesicle stage oocytes; Ne, PCR negative control. Amplification of *TUBULIN *is shown as an internal control.

We assessed the presence of transcripts for *MYST4 *and for all *MYST4 *isoforms simultaneously (*MORF*, *MORFα *and *MORFβ*) by RT-PCR analysis in different bovine tissue samples, including brain, thymus, muscle, lung, heart, liver, kidney, spleen, testis, ovary, uterus and oocyte. The three splicing variant sequences were similar except for the gap present in *MORF *and *MORFα *compared to *MYST4 *(Fig. 1B) which was restrictive for designing primers. Individual analysis using the different sets of primers for the splicing variants resulted in no or nonspecific amplification of *MORF *and *MORFα*, therefore they could not be investigated. Amplicons of expected sizes were generated for *MYST4 *and all-*MYST4*-iso, as well as for the housekeeping gene *TUBULIN *(Fig. 2). The mRNA transcripts for *MYST4 *and all-*MYST4*-iso were present in all tissues tested. Although this was not a quantitative analysis, the amplifications for the thymus, spleen, testis and uterus gave more intense bands with the same amount of cycles/cDNA compared to the other samples with both the *MYST4 *and all-*MYST4*-iso primers. However, the amplifications from the oocytes samples were amongst the most intense with the all-*MYST4*-iso primers, whereas they were ones of the lowest detected with the *MYST4 *primers.

### MYST4 Protein in Somatic Tissues

**Figure 3:**
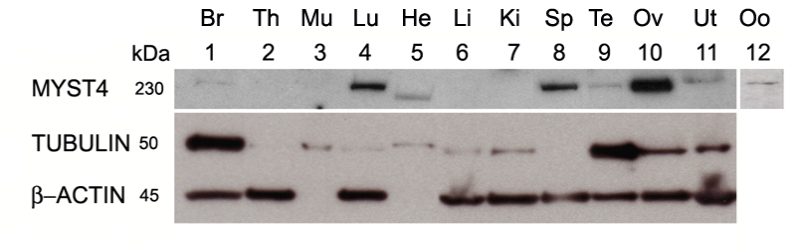
**Western blot analysis of MYST4 protein**. Protein extracts were subjected to SDS-PAGE and blotted onto nitrocellulose before being probed with an anti-bovine MYST4. From left to right, Br, brain; Th, thymus; Mu, muscle; Lu, Lung; He, heart; Li, liver; Ki, kidney; Sp, spleen; Te, testicle; Ov, ovary; Ut, uterus; Oo, germinal vesicle stage oocytes. TUBULIN and β-ACTIN antibodies were incubated simultaneously on the same membrane and were used as control.

In order to characterize the distribution of MYST4, an antibody was raised against a peptide specific only to MYST4 and its isoforms. The same tissues that were used for mRNA transcript analysis were subjected to Western blotting. As shown in Fig. 3, one positive protein band of about 230 kDa associated to MYST4 was predominantly present in the lung, spleen and ovary, whereas the same protein was also present but faintly detected in the brain, testis, uterus and oocytes. In the heart a single sharp band of about 200 kDa was detected. This protein is presumably the MORF isoform, which has a calculated molecular weight of 197 kDa. Peptide-blocking assays established that our MYST4 antibody was specific for the MYST4 peptide used for antibody production since the single distinct band disappeared (not shown). β-ACTIN was used as a loading control, but since some tissue extracts lacked this specific isoform, the samples were additionally probed with an anti-TUBULIN antibody.

### MYST4 Immunohistochemistry in Ovary

**Figure 4:**
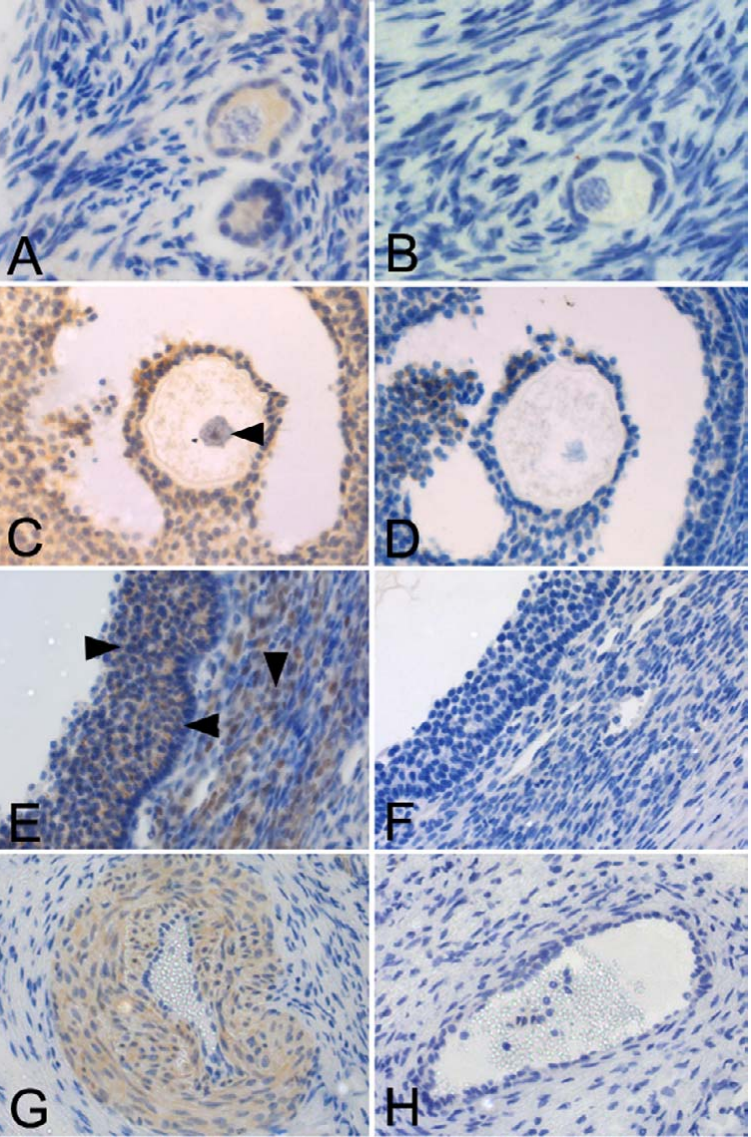
**Ovarian localization of MYST4 protein by immunohistochemistry**. Ovarian sections showing: primordial follicles (A, B); antral stage follicle, arrowhead showing oocyte nuclei (C, D); portion of large antral follicles, arrowheads indicating granulosa cells (right), theca (down) and basal lamina (left) (E, F) and blood vessels (G, H). Positive sections were incubated with anti-MYST4 (A, C, E, G,) and negatives were prepared by peptide-blocking assay (B, D, F, H). Original magnifications: 200× (C, D, E, F, G, H) and 400× (A, B).

Since our Western blot experiments revealed that only a portion of the strong MYST4 signal in the ovary was associated with oocytes, immunolocalization experiments were performed on ovary sections to distinguish which other cell types expressed MYST4. In these tissue sections, MYST4 expression was confined to follicles and to blood vessels, as there was no other signal within the stroma. A closer look at primordial follicles (Fig. 4A) revealed that MYST4 was intensely expressed in the cytoplasm of the oocyte, whereas the nucleus remained negative. MYST4 was also present in the cytoplasm of the simple squamous epithelium layer surrounding the oocyte, while cells in the proximity of the follicle wall were negative. In larger follicles, like those at the antral stage, granulosa cells adjacent to the oocyte and those covering the inside of the follicle strongly expressed MYST4 (Fig. 4C). The oocytes contained within these follicles displayed MYST4 in both their cytoplasm and nucleus (Fig. 4C). In large follicles MYST4 was not only present in granulosa cells, but also on the other side of the basal lamina, in the theca cells (Fig. 4E). While essentially all granulosa cells expressed MYST4 in their cytoplasm, only a portion of theca cells expressed MYST4. Within the theca cell population positive for MYST4, the majority displayed a strong signal for this protein in their nucleus, however a faint signal was also observed in some cell cytoplasm. Additionally ovary sections revealed that cells composing blood vessels also expressed MYST4 in their cytoplasm (Fig. 4G).

### MYST4 mRNA Profiles in Oocytes and Embryos

**Figure 5:**
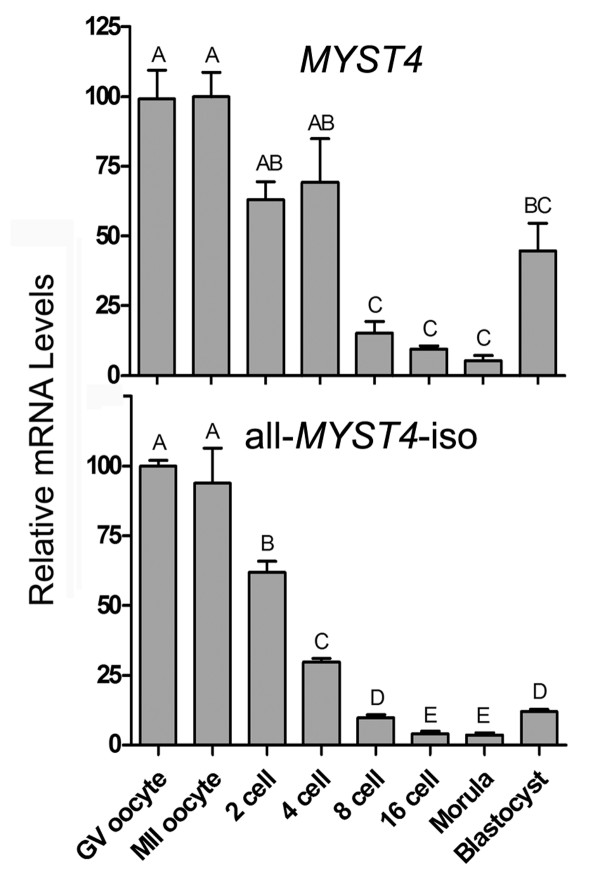
**Quantification of *MYST4 *and all-*MYST4*-iso in bovine oocytes and early embryos using real-time RT-PCR**. Each developmental stage was analyzed in triplicate using 0.5 oocyte or embryo per reaction. The relative mRNA levels shown represent the quantity of transcript corrected for the *GFP *value obtained for each pool. The highest level was attributed the relative value of 100. Shown is the relative mRNA abundance (mean ± SEM). Different letters indicate a significant difference of relative mRNA abundance (P < 0.05).

The mRNA profiles for *MYST4 *and all *MYST4 *isoforms (simultaneously) were assessed in oocytes and throughout bovine *in vitro *embryo culture by quantitative RT-PCR. The *MYST4 *variant and all-*MYST4*-iso exhibited a similar overall mRNA profile in oocytes and throughout early embryo development (Fig. 5). The highest relative levels of *MYST4 *mRNA were found in immature GV and mature MII oocytes. Transcript levels measured in the oocyte stages as well as in the 2- and 4-cell stages did not differ significantly, but a significant reduction was observed in the 8-cell embryos. The relative mRNA levels were lowest in 8-cell, 16-cell and morula embryos but tended to increase in blastocysts (Fig. 5A).

The relative levels of all-*MYST4*-isoforms were also at their highest intensity in both oocyte stages studied (Fig. 5B). Thereafter, significant decreases were observed in 2-, 4-, 8- and 16-cell embryos. The transcript levels for the combined isoforms were lowest in 16-cell and morula embryos. These minimal levels were subsequently followed by a significant increase in transcripts at the blastocyst stage.

### MYST4 Immunocytochemistry in Oocytes and Embryos

**Figure 6:**
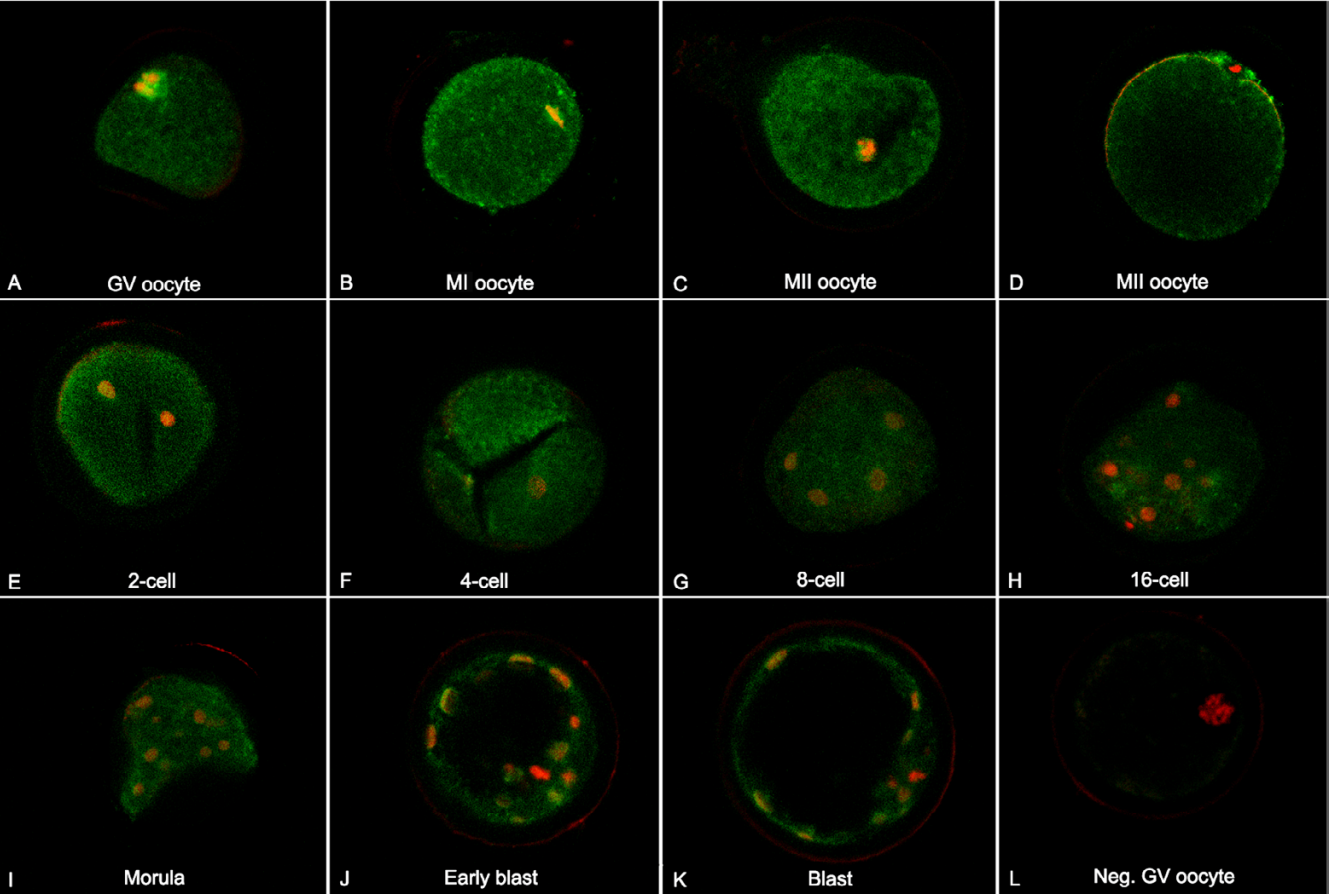
**Localization of MYST4 in oocyte and early embryo development**. Confocal representation of oocytes (GV, MI and MII) or embryos (2-, 4-, 8-, 16-cell, morula, early blastocyst and blastocyst) stained with an anti-MYST4 antibody (green signal) and with propidium iodide (red signal) to visualize the DNA. Original magnification 600×.

To evaluate the localization of MYST4 protein in bovine oocyte and embryo, immunolocalization experiments were performed on unfertilized bovine oocyte and throughout *in vitro *embryo development (Fig. 6). The immature oocyte strongly expressed MYST4 in its germinal vesicle (oocyte nucleus) and uniformly in its ooplasm (Fig. 6A). As the oocyte resumes its maturation, the GV breaks down (GVBD) and no nuclear membrane is noticeable at the metaphase I (MI) stage of meiosis. At that moment, MYST4 was not present on the chromatin but appeared to concentrate on the mitotic spindle and scattered in the ooplasm (Fig. 6B). In the *in vitro *matured oocyte (MII), MYST4 remained present throughout the ooplasm and along condensed chromosomes (Fig. 6C), but the first expulsed polar body did not reveal any staining (Fig. 6D). During the subsequent cleavage stages, in 2-, 4-, 8- and 16-cell embryos, MYST4 was clearly expressed in the cytoplasm (Fig. 6E, F, G and 6H). However, nuclear localization during that period in blastomeres was not as obvious compared to GV oocytes. Compact morula embryos exhibited a similar MYST4 expression profile in the cytoplasm and nucleus (Fig. 6I). In early and expanding blastocysts, MYST4 was observed in both the cytoplasm and nucleus of cells composing the trophectoderm and inner cell mass (Fig. 6J and 6K). However, the staining intensities of the nuclei were not similar, with some showing strong signal whereas it was weaker or absent in others (Fig. 6J and 6K). For all stages, negative controls were performed with a peptide-blocking assay, of which an example is shown (Fig. 6L).

### MYST4 Immunohistochemistry in Testis

**Figure 7:**
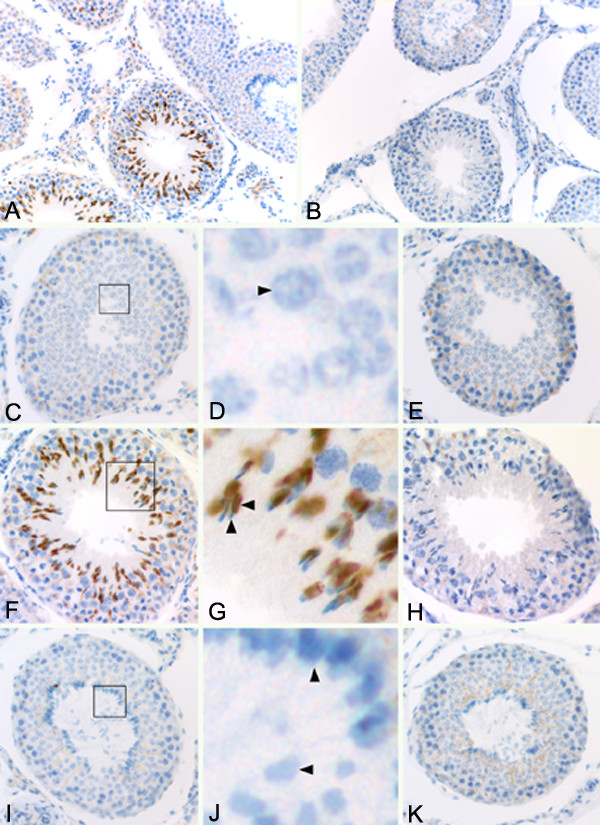
**Testicular localization of MYST4 protein by immunohistochemistry**. Testicular sections showing seminiferous tubules (A, B), containing mostly: primary spermatocytes and round spermatids (C, D, E), round spermatids and elongating spermatids (F, G, H), round spermatids and elongated spermatids (I, J, K). Images D, G, K are enlargements of boxed sections in C, F, I respectively. In magnified sections, arrowheads indicate: round spermatocyte (D), nucleus (left) and tail (up) of an elongating spermatid (G) and nuclei of elongated spermatids located in inner (up) wall and inside (left) of the lumen (J). Positive sections were incubated with anti-MYST4 (A, C, D, F, G, I, J) and negatives were prepared by peptide-blocking assay (B, E, H, K). Original magnification 1000× (A, B).

To establish if MYST4 expression in testis could also be restricted to specific cell types, testis sections were immunostained with the MYST4 antibody (Fig. 7). In the first cross-section (Fig. 7A), MYST4 was only present in some seminiferous tubules whereas others did not show any staining. This strongly suggests that its expression is developmentally regulated and is stage-specific during spermatid differentiation (spermiogenesis). MYST4 appeared to localize in cells facing the lumen of the seminiferous tubules and was cell-specific during spermiogenesis. To investigate this hypothesis, seminiferous tubules at different stages of spermatogenesis were closely observed (Fig. 7C to 7K). The spermatid differentiation process in bovine is divided in 14 described steps that include round (steps 1–7), elongating (steps 8–12) or elongated spermatids (steps 12–14) [21]. These various spermatids, from steps 1–12, are distributed in a specific manner within the seminiferous tubules (additional file 1). The seminiferous tubules are also divided into classes, called stages (I to XII), accordingly to the seminiferous epithelium cycle. Additionally, diverse types of germ cells (spermatogonia and spermatocytes) are distributed inside the different stages of the seminiferous epithelium cycle [21]. In seminiferous tubules containing primary spermatocytes and round spermatids (Fig. 7C &7D), which correspond to stage VII of the seminiferous epithelium cycle, no MYST4 was detected. However, in seminiferous tubules cross-sections at stages VIII to XI, intense staining of the MYST4 protein was detected in elongating spermatids (Fig. 7F). At higher magnification, the image clearly revealed that only the heads (nucleus), and not the mid-sections, of elongating spermatids were stained by MYST4 antibody (Fig. 7G). In this section (Fig. 7F and 7G), MYST4 immunolocalization was clearly restricted to elongating spermatids, as no spermatocytes were stained. Further along spermatogenesis, in stages IV to VI of the seminiferous epithelium cycle, the elongated spermatids displayed no immunoreactivity to MYST4 (Fig. 7I). The elongated spermatid nuclei located on the inner wall facing the lumen as well as those inside the lumen were negative for MYST4 (Fig. 7J).

## Discussion

As a result of its unique structural domain organization as well as its presence in various multisubunit complexes [11], MYST4 is probably involved in the regulation of a variety of biological processes. Although it is established that MYST4 is a potent histone H3 and H4 acetylase [12], the functional consequences of this enzymatic activity remain unknown. Its specific function as a HAT remains ambiguous, but its involvement in chromosomal abnormalities found in leukemia patients [14,15,22] and its normal presence in neurogenesis development have already been established [18,23]. In this paper we aimed to characterize *MYST4 *and the combination of all *MYST4 *isoforms simultaneously (*MORF, MORFα, MORFβ*) in various tissues, but more specifically in reproductive tissues and in gametes.

Based on its sequence, the bovine MYST4 protein is highly similar to its human counterpart, with almost 95% homology. Bovine MYST4 contains the same structural domains that make MYST4 so unique and therefore we can assume that bovine and human MYST4 take part in the same cellular processes.

### Expression of MYST4

A previous report revealed that human *MORF *is ubiquitously expressed in somatic tissues [12]. Transcript levels were high in tissues like heart, pancreas, testis and ovary, whereas they were barely detectable in the lung. In our study, we determined that the mRNA transcripts for the *MYST4 *gene as well as for all *MYST4 *isoforms were also ubiquitously present in all tissues tested, with different intensities.

Because the mRNA transcripts for the different splicing variants were present in each tissue tested, we thought they would parallel the protein levels. However, MYST4 protein detection was quite variable in the same tissues, ranging from high (lung, spleen and ovary) to undetectable (thymus, muscle, liver and kidney). Surprisingly, only one of the tissues (heart) revealed a specific band with a different molecular weight compared to the other tissues. Protein extracts from the heart displayed only one band which may be the shortest isoform, MORF.

### MYST4 in female gametogenesis

Oocytes also expressed the MYST4 protein, albeit weakly, and thus could not account for the important levels found in the ovary extracts, suggesting that other cell types could express MYST4. Immunolocalization on ovary sections confirmed this by revealing that MYST4 was expressed in follicular cells (granulosa and theca) enclosing the oocyte, as well as in blood vessels. Although this is the first time that the MYST4 acetylase is associated with folliculogenesis, various acetylation events occur within granulosa and theca cells during follicle formation and growth. Numerous experiments using ChIP (Chromatin immunoprecipitation) assays on these cells revealed that specific histone acetylation events in the promoter region of ovarian genes are affected by the increase in reproductive hormones (reviewed in [24]). *In vitro *culture of rat granulosa cells treated with FSH (follicle-stimulating hormone) enhances the transcriptional activation of the *SGK *(serum-glucocorticoid kinase) and the *FOS *(FBJ osteosarcoma oncogene) genes by increasing the acetylation of specific lysine residues on histone H3 [25]. In addition, granulosa cells obtained from monkeys treated with hCG and from human IVF (*in vitro *fertilization) patients also treated with hCG exhibited histone H3 acetylation in the regulatory regions of the *StAR *(steroidogenic acute regulatory protein) gene [26]. Furthermore, inhibition of HDACs in cultured human theca cells increased total histone H3 and H4 acetylation levels, along with mRNA transcription for CYP11A (cytochrome P450, family 11, subfamily A, polypeptide 1) and CYP17 (cytochrome P450, family 17) [27]. Given that the MYST4 proteins are restricted to certain specialized cells in the ovary and have a strong histone H3/H4 acetylation activity, they could regulate the transcription of specific genes during folliculogenesis. The functions of MYST4 in follicular cells may be somewhat distinctive since the protein is mainly nuclear in theca cells and cytoplasmic in granulosa cells. Dormant oocytes enclosed in primordial follicles also lacked MYST4 in their nucleus, but the oocytes contained in the larger follicles exhibited MYST4 in both cytoplasm and nucleus. Since dormant oocytes are deficient in transcription at this point [28] they may not require this HAT in their nucleus for regulatory processes, whereas growing oocytes are in a transcriptionally active state and thus necessitate enzymes responsible for chromatin remodeling.

Related mRNA profiles in oocytes and throughout embryo development were observed between *MYST4 *and all-*MYST4*-iso. However, since higher levels of transcripts were measured with the all-*MYST4*-iso in oocytes compared to the subsequent embryo stages, whereas *MYST4 *was equally present in the first four stages, we assume that the splicing variants *MORF *and/or *MORFα *are more abundant in the GV and MII oocytes compared to other embryo stages. Globally, a significant amount of all-*MYST4*-iso transcripts was lost following each embryo division up to the 16-cell stage, followed by a slight increase in mRNA levels between morula and blastocyst embryos. High levels of transcripts in oocytes could be mRNA accumulated during oocyte growth and translated into protein in subsequent stages of development until the embryo is able to produce its own mRNA at the MET (maternal embryonic transition). However the immunolocalization study, although not quantitative, revealed that similar intensity of staining for cytoplasmic MYST4 was found throughout development. This could indicate that the protein has an extensive half-life or the transcripts produced by post-MET embryos are constantly being translated into new proteins and do not accumulate. Interestingly, GV oocytes contained the largest measured levels of *MYST4 *transcripts and had the highest accumulation of the protein in its nucleus. Since there is still some active transcription in GV oocytes [29], MYST4 may potentially be implicated in this permissive chromatin state by co-regulating transcriptional complexes via its transcriptional activation domain. Surprisingly, during oocyte maturation when chromosomes are lined up along the equator at the metaphase-1 stage, MYST4 was located in the vicinity of the meiotic spindle rather than on chromosomes. Interaction with chromatin is expected, since MYST4 contains a H1/H5 domain that mediates self-association and interaction with core histones and the nucleosomes [11]. Because there is no nuclear membrane during MI in meiosis, there was no barrier to retain MYST4 at this location. Therefore it may be implicated in the meiotic spindle action to separate the chromosomes or in other related mechanisms. It is unlikely that it acetylates histones H3 and H4 at that point, since histones are globally deacetylated in oocytes during meiosis [30] and improper histone deacetylation in the course of meiosis is associated with aneuploidy and embryo death [31]. Alternatively, the α-TUBULIN proteins composing the microtubules could perhaps be a target for MYST4, since they are acetylated at different points in meiotic and mitotic divisions in mouse oocytes [32]; however, no studies have yet examined if MYST4 could acetylate α-TUBULIN. Although MYST4 appeared to be present in the nucleus from the 2-cell until the 16-cell stage embryo, no major accumulation was observed in the nucleus. In the morula and blastocyst stages, some nuclei appeared to have the same accumulation as that observed in oocytes. This nuclear accumulation may be associated to specific stages in the cell cycle and since there are more individual cells in those embryos, the chances of detecting this accumulation are increased. The presence of MYST4 in the nucleus also coincides with the activation of specific genes involved in the delineation of the blastocyst inner cell mass and trophectoderm [33].

### MYST4 in male gametogenesis

Since MYST4 was linked with reproductive cells in the ovary, we wondered if its presence in testis could have a similar function. Surprisingly, in this organ, MYST4 was restricted to specific cells. The antibody brightly stained only the nucleus of elongating spermatids found in stages VIII to XI of the seminiferous epithelium cycle, which normally enclose step-8 through -11 spermatids [21]. The spermatid differentiation process in bovine is divided in 14 described steps that include round (steps 1–7), elongating (steps 8–12) or elongated spermatids (steps 12–14) [21,34]. The adjacent seminiferous tubules of the same testis section, containing spermatids at different stages of development, were not immunoreactive. Because there are various nuclear modification events that occur during spermiogenesis, MYST4 may be a key player in one of them. In the nucleus of elongating spermatids at different stages, important acetylation processes occur to ensure proper histone displacement for their replacement by transition proteins and protamines [35-37]. During these processes, different residues of the core histones (H3, H4, H2A and H2B) are targets for acetylation. In mouse spermiogenesis, there are 16 reported steps, with steps 9 to 12 associated with the elongating spermatids [38]. Acetylation of histone H3 is limited to steps 10 and 11, whereas histone H4 is hyperacetylated from steps 8 to 11. For both H3 and H4, the intense staining seems homogeneously distributed over the nuclear region. Furthermore, the four potential sites of acetylation on histone H4 (Lys 5, 8, 12, and 16) were individually studied with specific antibodies and revealed to be all immunoreactive in steps 8 to 11 of the elongating spermatids [36].

Other acetylation events on histones H2A (steps 9 to 11) and H2B (steps 10 and 11) are also observed in elongating spermatids. To date, the only genes thought to be associated with histone H4 hyperacetylation in elongating spermatids prior to the histone-to-protamine exchange are all members of the Cdy (chromodomain protein, Y chromosome)-related family [38,39]. In the mouse, where two transcripts evolved for that gene, both *cdyl *(Cdy-like) and *cdyl2 *exhibit a ubiquitous, long transcript as well as a highly abundant, testis-specific short transcript [39]. In human, members include the *CDYL *and *CDYL2 *genes which are ubiquitously express in tissues, and *CDY *which is an abundant testis-specific gene [39]. *CDY *and its homologs encode a HAT that acetylates free histones H4 and H2A [38]. By immunolocalization, it was shown that mouse Cdyl is abundantly expressed in the nuclei of elongating spermatids at the time histone H4 is hyperacetylated [38]. Although our immunolocalization experiments were not performed on individual elongating step sections, we could clearly distinguish that MYST4 was also only present in elongating spermatids during spermatogenesis. Furthermore, MYST4 is not only able to acetylate free H2A, H3 and H4 when used as substrates, but can efficiently acetylate nucleosomal histones as well [12]. With the new data obtained in our study added to previous reports on MYST4, strong evidence connects this protein with H2A, H3 and H4 acetylation during spermiogenesis. However, because some of the histone modifications mentioned above are also present at some other stages like spermatogonia and preleptotene spermatocytes, histone acetylases other than those of the CDY and MYST4 families are likely to be involved in spermatogenesis.

### MYST4 *in vivo *characteristics

Although MYST4 was discovered several years ago, it has never been linked to the formation of a stable multisubunit HAT complex. Recently, MYST4 and its close relative MOZ (monocytic leukemia zinc finger) were characterized as part of a histone H3 specific HAT complex important for DNA replication [40]. This HAT complex also includes a member of the tumor suppressor family (ING5, inhibitor of growth 5), proteins usually associated with chromatin (BRPF 1/2/3) and an uncharacterized protein homologous to a subunit of a yeast acetyltransferase (Eaf6, Esa1-associated factor-6). Since this complex was only able to acetylate H3K14 in HeLa cells [40], it is unlikely to be involved in the acetylation of H4 in spermatids. However, transcripts from related BRFP family members are preferentially expressed within spermatocytes in the testis [41,42] and in oocytes [42], suggesting that BRFP proteins may be present and associated with MYST4 in other HAT complexes.

Although *MYST4 *knock-outs are not yet available to determine whether the gene is essential in reproduction or other important processes, a mutant mouse for its homolog *querkopf *exists. The homozygous *querkopf *mice display about 10% of the normal coding mRNA compared to wild type [18]. These mutant mice show craniofacial abnormalities, retarded postnatal growth, defects in the central nervous system [18] and severe problems in adult neurogenesis [23]. These physiological failures are caused by the essential function attributed to Querkopf in neural stem cells [18,43] and in bone formation through its involvement with RUNX1 [17,44]. Because there is still partial expression of wild-type *querkopf *mRNA, these mice are still able to develop despite all their defects. However for the moment, no information about other malformations or problems in folliculogenesis, spermatogenesis or embryo development was reported for the homozygous mutant mice. Even though MYST4 and MOZ interact in different protein complexes, they specifically regulate diverse stem cell populations [11,17,40]. Through its complete deletion in mice, Moz revealed itself as an essential regulator of hematopoietic stem cell maintenance [18,45]. Compared to the Querkopf or MYST4 mutants, MOZ-null mice die before or at birth.

## Conclusion

The work presented here reveals novel properties for the histone acetylase MYST4. The data and conclusions from bovine MYST4 will undoubtedly apply to the human protein since both sequences show extensive similarities. In addition to providing the first proteomic study of this poorly studied protein, this is the first report to link MYST4 with early gametogenesis. To our knowledge, MYST4 is the only HAT to be described in cells (elongating spermatids, oocyte, granulosa and theca cells) related to gamete formation in both male and female. The availability of a highly specific and functional antibody will now allow the use of techniques such as pull-down assay and chromatin immunoprecipitation, to identify protein complexes and isolate chromatin target sites associated with MYST4 in transcriptional regulation events in reproductive tissues and other specific cell types. This antibody will also provide a new tool to study the implication of MYST4 in acute myeloid leukemia (AML).

## Methods

Unless otherwise stated, all materials were obtained from Sigma-Aldrich (St. Louis, MO).

### Oocyte Recovery and *In Vitro *Embryo Production

The procedures for oocyte recovery and *in vitro *embryo production have been described previously [46]. Using this culture system, more than 30% of inseminated oocytes routinely reached the blastocyst stage. Briefly, cumulus-oocyte complexes (COCs) were recovered from bovine slaughterhouse ovaries, matured in modified synthetic oviduct fluid (SOF) for 24 hrs, then transferred and fertilized in a modified Tyrode lactate medium. Following fertilization, putative zygotes were mechanically stripped of their cumulus cells, washed in PBS and transferred to modified SOF medium for embryo development. The 2-, 4-, 8-, and 16-cell embryos were collected at 36, 48, 72, and 108 hrs post-insemination, respectively, whereas morulae and blastocysts were collected after 6 and 8 d of development. At each stage, all embryos were washed 3 times in PBS, collected in pools of 20, frozen and stored at -80°C until RNA extraction. All oocyte and embryo pools used for RNA extractions were collected and analyzed in triplicates.

### RNA Extraction and cDNA Preparation

For oocyte and embryo RNA extraction and cDNA preparation, *GFP *RNA was used as an external control [47,48]. Ten pg of exogenous *GFP *RNA containing a poly-A tail [46] was added to each pool of oocytes and embryos prior to RNA extraction. RNA extractions of the oocyte or embryo pools containing *GFP *RNA were then performed using the PicoPure RNA isolation kit (Arcturus/Molecular Devices Corporation, Sunnyvale, CA) and directly used for cDNA preparation as previously described [46].

Total RNA from bovine tissues was extracted using TriZol Reagent (Invitrogen, Burlington, ON, Canada) according to the manufacturer's protocol. All RNA extracts used were treated with RNase-free DNase (Promega, Nepean, ON, Canada). cDNA synthesis was performed using Omniscript reverse transcriptase (Qiagen, Mississauga, ON, Canada) with 600 ng of total RNA and oligo-d(T)_12–18_, as described by the manufacturer.

### Polymerase Chain Reaction (PCR)

Primers for all the genes analyzed are listed in Table 1 and were designed using bovine sequences when possible, or from a consensus derived from human and mouse sequences. The primers for *MYST4 *were designed in a region specific to this variant (Fig. 1B), whereas the primers for all-*MYST4*-iso were designed in a downstream area common to all variants (not shown). Real-time PCR was performed on a Lightcycler apparatus (Roche Diagnostics, Laval, QC, Canada) using SYBR Green incorporation as previously described [47]. Briefly, for each gene, a standard curve consisting of purified PCR product was included in the run. Each of the PCR reactions was performed using the equivalent of 0.5 oocyte/embryo. The annealing temperatures for each set of primers are listed in Table 1. The PCR products were separated by agarose gel electrophoresis and sequenced. *GFP *mRNA was used as an external control to account for experimental errors due to techniques involved or materials used for RNA extraction and reverse transcription [46,47].

**Table 1:** Primers used for RT-PCR experiments.

**Gene**	**Primer sequences**	**Accession numbers**	**Length of PCR products (bp)**	**T**_**M **_**(°C)**
*MYST4 *(complete sequence)	Up 5'-ATGTTCTTTCACCCGAATGC-3'	AF119231	6590	55
	Low 5'-AAATAAATGGTGCCAACAAATG-3'	NM_017479		
*MYST4*	Up 5'-AGTTCACAAAAGTCCAGCAC-3'	AF119231	300	60
	Low 5'-GCTAGAGGAGGAGGAGTGAG-3'	XM_880782		
all-*MYST4*-iso	Up 5'-*ACCTTCAGCCTTGCCAAACT*G-3'	AF119231	296	60
	Low 5'-TCTTTGGCTGTGAGAGATGC-3'			
*TUBULIN*	Up 5'-CTCTGCTGAGAAAGCCTACCA-3'	BC018948	400	60
	Low 5'-CCACGTACCAGTGAACAAAGG-3'			

Standard PCR reactions (*MYST4*, all-*MYST4*-iso and *TUBULIN*) were performed using cDNA equivalent to 1 oocyte or 1 μl of first strand cDNA for somatic tissues, and *Taq *Gold polymerase (Applied Biosystems, Streetsville, ON, Canada) for 35 cycles as described by the manufacturer. For PCR amplification of the *MYST4 *complete sequence, the cDNA equivalent to 5 oocytes was used with Taq Advantage 2 (Clontech, Mountain View, CA) following the manufacturer's indications.

### Western Blotting

Proteins from brain, thymus, muscle, lung, heart, liver, kidney, spleen, testis, ovary, and uterus were extracted on ice in triple lysis buffer (50 mM Tris-HCl pH 7.6, 150 mM NaCl, 0.02% NaN_3_, 0.1% SDS, 1% NP40, 0.5 % deoxycholic acid and protease inhibitors [Roche]) as previously described [47]. Bovine oocytes (n = 100) were denuded, washed in PBS then frozen at -80°C. Fifteen μg of each extract or 100 oocytes were lysed in 2× SDS loading buffer containing 6% β-mercaptoethanol at 95°C for 5 min. Proteins were resolved on standard 6% SDS-PAGE gels and transferred onto nitrocellulose membranes (Osmonics, Minnetonka, MN) using a semi-dry transfer apparatus (BioRad, Hercules, CA). Blotted membranes were blocked in TBST (25 mM Tris-HCl pH 7.6, 125 mM NaCl and 0.1% Tween-20) containing 5% ECL Advance Blocking Agent (Amersham Biosciences, Piscataway, NJ) for 1 hr at room temperature. Membranes were then incubated with a 1:7500 dilution of MYST4 antibody with 3% ECL Advance Blocking Agent in TBST overnight at 4°C. This purified anti-MYST4 was raised in rabbits against a 15-mer KLH-conjugated peptide (YGGLDGKGAPKYPSC) which is specific to bovine MYST4 (AgriSera AB, Vännäs, Sweden). The membranes were washed 1 × 15 min and 4 × 5 min in TBST, then incubated with a peroxidase-conjugated antibody (Molecular Probes, Burlington, ON, Canada) diluted 1:200,000 in TBST-3% ECL Advance Blocking Agent for 45 min. Finally, membranes were washed 4 × 5 min in TBST followed by a 1 × 15 min wash in TBS before the chemiluminescent signal was revealed using ECL Advance Reagent (Amersham). To assess anti-MYST4 specificity, a peptide-blocking assay was carried out following the manufacturer's recommendations. The same protocol was used with the β-ACTIN antibody (Cell Signaling Technology Inc., Danvers, MA) and the α-TUBULIN antibody (Sigma). Briefly, 15 μg of each extract were resolved on a 15% gel. A 1:10,000 dilution of β-ACTIN and 1:250,000 of α-TUBULIN antibodies were simultaneously incubated with the blotted membrane before being probed with the peroxidase-conjugated secondary antibody.

### Immunocytochemistry

Oocytes and embryos used for the immunocytochemistry experiments were obtained using the same method mentioned above. Immature germinal vesicle (GV) oocytes and mature metaphase II (MII) oocytes, 1-, 2-, 4-, 8-, 16-cell embryos, morulae and blastocysts, were fixed and permeabilized on poly-lysine slides as previously described [47] using 2% paraformaldehyde for 30 min at RT. After blocking in TBST-5% milk, the MYST4 antibody (1:1000 in wash solution containing 3% dry milk) was added. The cells were subsequently washed and incubated with a fluorescein-conjugated goat anti-rabbit IgG (Molecular Probes) diluted 1:1000 in wash solution with 3% dry milk, washed again and incubated with propidium iodide in PBS (final concentration of 10 μg/mL) for 10 min. Negative controls were prepared with either the fluorescein-conjugated goat anti-rabbit IgG or with pre-immune serum derived from the same rabbit that produced the anti-MYST4. To assess anti-MYST4 specificity in oocytes and embryos, a peptide-blocking assay was carried out following the manufacturer's recommendations. The fluorescein-conjugated goat anti-rabbit IgG controls were used to set the background fluorescence.

### Immunohistochemistry

Testis and ovary samples were fixed in Bouin fixation solution and mounted in paraffin blocks as described previously [49]. Briefly, non-specific sites were blocked with 1% BSA in PBS. The slides were incubated for 2 hrs at RT in the presence of anti-MYST4 (1:500), and tissue sections were then covered with a goat anti-mouse IgG coupled to biotin for 1 hr at RT. After carefully washing the slide, streptavidin-HRP was deposited on tissues for 30 min at room temperature and the immune complex was then revealed using 3,3'-diaminobenzidine (DAB). The slides were mounted in mowiol (Calbiochem, La Jolla, CA) and observed by light microscopy. For both ovary and testis, negative controls were carried out by peptide-blocking assay following the manufacturer's recommendations.

### Statistical Analysis

The level of mRNA for each gene subjected to statistical analysis was normalized using the *GFP *external control [46-48]. The value obtained for each gene, within each pool of cDNA, was divided by the value obtained for GFP in the same cDNA pool. Data are presented as mean ± SEM. Statistically significant differences in mRNA levels between each developmental stage were calculated by protected ANOVA (SAS Institute, Cary, NC), and treatment and replicate were included in the model. Differences were considered statistically significant at the 95% confidence level (P < 0.05).

## Authors' contributions

SM designed the experiments, performed most experimental work and wrote the manuscript. GM carried out the immunohistochemistry. CV was involved in the biological sample collection, immunocytochemistry and statistical analysis. PL participated in the interpretation of the immunohistochemistry results. MAS supervised the study and participated in the design of experiments. All authors reviewed and approved the final manuscript.
